# First report of teclistamab in a patient with relapsed AL amyloidosis and multiple myeloma

**DOI:** 10.1002/jha2.772

**Published:** 2023-08-21

**Authors:** Nelson Leung, Jennifer A Chapman, Sumeet Bhatia

**Affiliations:** ^1^ Division of Nephrology and Hypertension Mayo Clinic Rochester Minnesota USA; ^2^ Division of Hematology Mayo Clinic Rochester Minnesota USA; ^3^ Community Health Network MD Anderson Indianapolis Indiana USA

**Keywords:** AL amyloidosis, bispecific T‐cell engager, IgD, multiple myeloma, teclistamab

1

Immunoglobulin light chain (AL) amyloidosis is a consequence of monoclonal gammopathy that can occur with, but more commonly, without multiple myeloma (MM) [1, 2]. Patients with AL amyloidosis have benefitted significantly from the tremendous progress made in the treatment of MM especially with the addition of anti‐CD38 antibodies to novel agents [3, 4, 5, 6, 7]. Despite that, it is important to keep in mind that the risk of adverse events (AEs) is different in patients with AL amyloidosis due to organ injuries that render them less tolerable to certain therapies [8, 9]. B‐cell maturation antigen (BCMA) is one of the newest targets in the treatment of MM. Several therapies targeting BCMA with chimeric antigen receptor (CAR) T‐cell, antibody drug conjugates (ADC) and bispecific T cell engagers have already been approved by the U.S. Food and Drug Administration (FDA) for patients with relapsed or refractory MM [10, 11, 12, 13]. BCMA targeting ADC and CAR T‐cell therapy have been reported for patients with AL amyloidosis [14, 15]. We would like to report the first use of teclistamab, a bispecific CD3 T‐cell engager targeting BCMA, in a patient with AL amyloidosis and multiple myeloma.

Patient is a 76‐year‐old female who presented at age 71 with a positive fat aspirate for amyloid. She had developed bilateral carpel tunnel syndrome at age 66 and underwent bilateral carpel tunnel release. Her symptoms returned after surgery and were refractory to prednisone requiring secukinumab. She developed dyspnea on exertion and lower extremity edema 1 year prior and macroglossia, constipation and easily bruising appeared 3 to 4 months prior to diagnosis. The amyloid deposits on the fat aspirate were not typed. A M‐spike was not detectable but immunofixation found a monoclonal IgD lambda, a free monoclonal lambda free light chain (FLC) and an IgG kappa. Serum kappa FLC was 1.95 mg/L, lambda FLC was 825 mg/L with a kappa to lambda ratio of 0.0024 and a differece of involved to uninvolved (d)FLC of 823 mg/dL. Bone marrow biopsy showed 30% involvement with a lambda light chain restricted plasma cell clone. Myeloma FISH was pertinent for deletion TP53, monosomy 13 and trisomy 3, 7, and 15. Echocardiogram showed a left ventricular ejection fraction of 55%, ventricular septal thickness of 10 mm and global average left ventricular longitudinal peak systolic strain of −17% (normal < −18%). Fifth generation cardiac troponin T was 126 ng/L and N‐terminal pro brain natriuretic peptide (NTproBNP) was 4323 pg/ml. Serum creatinine was 107 μmol/L and 24‐h urine was 1.3 g/d. A biopsy of a subcutaneous nodule near her wrist showed (lambda light chain) amyloid by mass spectrometry. She had Mayo stage 3B by modified European criteria and Mayo stage IV by Mayo 2012 criteria, renal stage II AL amyloidosis. Her performance status by Karnofsky was 70 and was 1 by ECOG. CT skeletal survey revealed multiple lytic lesions in the axial skeleton.

Patient was enrolled in a clinical trial MC1382 with ixazomib‐cyclophosphamide‐dexamethasone for her front‐line therapy (Table 1). She had an excellent response achieving minimal residue disease (MRD) negativity by next generation flow to a sensitivity of 10^−5^. After 12 months of therapy, ixazomib maintenance was started but her disease relapsed 6 months into maintenance therapy. Therapy was switched to daratumumab‐pomalidomide‐dexamethasone. Unfortunately, she developed severe fever and chills during daratumumab infusion and daratumumab was discontinued. Carfilzomib‐pomalidomide‐dexamethasone was initiated. She achieved a stringent complete response (SCR). NTproBNP decreased to 554 pg/ml and proteinuria normalized (Table 1). After 1 year, she developed peripheral neuropathy, neurologic spells, and severe fatigue. Symptoms improved with discontinuation of carfilzomib, and she was maintained on pomalidomide. Her disease again progressed after 9 months, and isatuximab‐carfilzomib‐dexamethasone was started. She achieved another SCR after one cycle and tolerated isatuximab without any adverse effects but her disease progressed after 1 year. Selinexor‐bortezomib‐dexamethasone was then administered for 3 cycles with no response. Serum kappa FLC was 1.4 mg/L, lambda FLC was 266 mg/L with a kappa to lambda ratio of 0.0053 and a dFLC of 265 mg/L. Creatinine was 109 μmol/L, cardiac troponin T was 15 ng/L, and NTproBNP was 2330 pg/ml. Decision was made to start teclistamab. Patient underwent standard dose escalation protocol and experienced no cytokine release syndrome (CRS) or neurotoxicity. Patient achieved another SCR with a kappa FLC of 7 mg/dL, lambda of 18 mg/dL and a dFLC of 11 mg/dL after two cycles and remains on therapy having completed six cycles.

**TABLE 1:** Lines of therapy and the corresponding response and adverse effects

Regimen	Response	Duration	Adverse effects	Proteinuria (mg/d)	NTproBNP (pg/ml)	Outcome
Ixazomib/Cyclophosphamide/Dexamethasone	MRD(‐)	18 m	None	2072 (pre) 238 (B)	4323 (pre) 554 (B)	Progressed on ixazomib maintenance
Daratumumab/Pomalidomide/dexamethasone	N/A	First cycle	Fever and chills	420 (pre)	722 (pre)	Daratumumab was discontinued
Carfilzomib/Pomalidomide/dexamethasone	SCR	12 m	Hair loss, neurologic spells, fatigue, neuropathy	420 (pre) 257 (B)	722 (pre)	Carfilzomib was discontinued
Pomalidomide dexamethasone	SCR	9 m	None	242 (pre)	3641 (pre) 1185 (B)	progressed
Isatuximab/Carfilzomib/Dexamethasone	SCR	12	None	242 (pre) 133 (B)	1185 (pre)	Progressed after 12 m
Selinexor/Bortezomib/Dexamethasone	NR	3 m		133 (pre)	1198 (pre)	
Teclistamab	SCR	3 m	None	225 (pre) 140 (B)	2330 (pre) 790 (B)	Therapy is ongoing

Abbreviations: (B), best value; MRD, minimal residue disease; NR, no response; (pre), value prior to treatment; SCR, stringent complete response.

Teclistamab is the first BCMA targeting bispecific T‐cell engager approved for treatment of relapsed or refractory MM [12]. It is currently indicated for MM patients who had at least 4 prior lines of therapy. In the pivotal phase 1–2 portion of MajesTEC‐1 trial, Teclistamab achieved an overall response rate of 63% in a heavily treated population (median prior lines of therapy = 5), where 77.6% were triple class refractory and 30.3% were penta‐refractory. Very good partial response or better was achieved in 58.8% while complete response or better was achieved in 39.4%. AEs grade 3 or higher were reported in 94.5% with the most common being neutropenia (64.2%), anemia (37.0%), and thrombocytopenia (21.2%). CRS was reported in 72.1% but only 0.6% were grade 3 or higher. Treatment with tocilizumab, glucocorticoids, or supplemental oxygen by nasal canula were provided in 36.4%, 8.5%, and 12.7% of the patients, respectively. Neurologic AEs were reported in 14.5% and with only 1 (0.6%) seizure in a patient with bacterial meningitis. Nine neurological AEs were documented in five patients. Although these rates are less than those reported in the CAR‐T cell trials, they are higher than other therapies for AL. Toxicity such as CRS and neurological AEs have been a concern in patients with AL. On the other hand, AL amyloidosis like multiple myeloma is not a curable disease and as patients will need access to newer agents with novel therapeutic targets. Successful use of ADC and CAR T‐cell therapy has been reported, which had led to clinical trials in relapsed or refractory AL amyloidosis patients [14, 15]. Our case shows for the first time that a patient with relapsed AL amyloidosis with heart, kidney, and soft tissue involvement and MM can be successfully treated with teclistamab. Hopefully, this will raise the awareness and interest of future clinical trials with teclistamab in relapsed or refractory AL amyloidosis.

## AUTHOR CONTRIBUTIONS

NL gathered, analyzed, and wrote the paper. JC and SB gathered the data.

## ETHICS STATEMENT

The study was exempt by the Mayo Clinic IRB.

## CONFLICT OF INTEREST STATEMENT

NL has stocks in AbbVie and a grant from Omeros. Other authors have no conflict of interest to declare.

## PATIENT CONSENT STATEMENT

Patient provided a written consent to publish her data.

## References

[1] Sidana S , Larson DP , Greipp PT , He R , Mcphail ED , Dispenzieri A , et al. IgM AL amyloidosis: delineating disease biology and outcomes with clinical, genomic and bone marrow morphological features. Leukemia. 2020;34(5):1373–82. 31780812 10.1038/s41375-019-0667-6 PMC8019395

[2] Kyle RA , Gertz MA . Primary systemic amyloidosis: clinical and laboratory features in 474 cases. Semin Hematol. 1995;32(1):45–59. 7878478

[3] Al Hamed R, Bazarbachi AH , Bazarbachi A , Malard F , Harousseau JL , Mohty M . Comprehensive Review of AL amyloidosis: some practical recommendations. Blood Cancer J. 2021;11(5):97. 34006856 10.1038/s41408-021-00486-4 PMC8130794

[4] Facon T , Kumar S , Plesner T , Orlowski RZ , Moreau P , Bahlis N , et al. Daratumumab plus lenalidomide and dexamethasone for untreated myeloma. N Engl J Med. 2019;380(22):2104–15. 31141632 10.1056/NEJMoa1817249 PMC10045721

[5] Kastritis E , Palladini G , Minnema MC , Wechalekar AD , Jaccard A , Lee HC , et al. Daratumumab‐based treatment for immunoglobulin light‐chain amyloidosis. N Engl J Med. 2021;385(1):46–58. 34192431 10.1056/NEJMoa2028631

[6] Moreau P , Attal M , Hulin C , Arnulf B , Belhadj K , Benboubker L , et al. Bortezomib, thalidomide, and dexamethasone with or without daratumumab before and after autologous stem‐cell transplantation for newly diagnosed multiple myeloma (CASSIOPEIA): a randomised, open‐label, phase 3 study. Lancet. 2019;394(10192):29–38. 31171419 10.1016/S0140-6736(19)31240-1

[7] Voorhees PM , Kaufman JL , Laubach J , Sborov DW , Reeves B , Rodriguez C , et al. Daratumumab, lenalidomide, bortezomib, and dexamethasone for transplant‐eligible newly diagnosed multiple myeloma: the GRIFFIN trial. Blood. 2020;136(8):936–45. 32325490 10.1182/blood.2020005288 PMC7441167

[8] Sanchorawala V , Wright DG , Rosenzweig M , Finn KT , Fennessey S , Zeldis JB , et al. Lenalidomide and dexamethasone in the treatment of AL amyloidosis: results of a phase 2 trial. Blood. 2007;109(2):492–6. 16960148 10.1182/blood-2006-07-030544

[9] Gertz MA , Lacy MQ , Dispenzieri A , Kumar SK , Buadi FK , Dingli D , et al. Trends in day 100 and 2‐year survival after auto‐SCT for AL amyloidosis: outcomes before and after 2006. Bone Marrow Transplant. 2011;46(7):970–5. 20935685 10.1038/bmt.2010.234

[10] San‐Miguel J , Dhakal B , Yong K , Spencer A , Anguille S , Mateos M‐V , et al. Cilta‐cel or standard care in lenalidomide‐refractory multiple myeloma. N Engl J Med. 2023;389:335–47. 37272512 10.1056/NEJMoa2303379

[11] Munshi NC , Anderson LD , Shah N , Madduri D , Berdeja J , Lonial S , et al. Idecabtagene vicleucel in relapsed and refractory multiple myeloma. N Engl J Med. 2021;384(8):705–16. 33626253 10.1056/NEJMoa2024850

[12] Moreau P , Garfall AL , Van De Donk NWCJ , Nahi H , San‐Miguel JF , Oriol A , et al. Teclistamab in relapsed or refractory multiple myeloma. N Engl J Med. 2022;387(6):495–505. 35661166 10.1056/NEJMoa2203478 PMC10587778

[13] Baines AC , Ershler R , Kanapuru B , Xu Q , Shen G , Li L , et al. FDA approval summary: belantamab mafodotin for patients with relapsed or refractory multiple myeloma. Clin Cancer Res. 2022;28(21):4629–33. 35736811 10.1158/1078-0432.CCR-22-0618 PMC9633344

[14] Zhang Y , Godara A , Pan S , Toskic D , Mann H , Sborov D , et al. Belantamab mafodotin in patients with relapsed/refractory AL amyloidosis with myeloma. Ann Hematol. 2022;101(9):2119–21. 35821344 10.1007/s00277-022-04890-z

[15] Oliver‐Caldes A , Jiménez R , Español‐Rego M , Cibeira MT , Ortiz‐Maldonado V , Quintana LF , et al. First report of CART treatment in AL amyloidosis and relapsed/refractory multiple myeloma. J Immunother Cancer. 2021;9(12):e003783. 34876408 10.1136/jitc-2021-003783 PMC8655576

